# Pre-extubation calculated serum osmolality and early invasive reintubation in critically ill adults: a dual-database study with multicenter external replication

**DOI:** 10.3389/fmed.2026.1984164

**Published:** 2026-10-21

**Authors:** Weibin Huang, Jiaxiang Bian, Youli Chen, Baolin Huang, Chuling Liu, Cunrong Chen

**Affiliations:** 1 Department of Critical Care Medicine, Fujian Medical University Union Hospital, Fuzhou, Fujian, China; 2 Department of Critical Care Medicine, Quanzhou First Hospital Affiliated to Fujian Medical University, Quanzhou, Fujian, China

**Keywords:** airway extubation, calculated serum osmolality, mechanical ventilation, metabolic phenotype, reintubation

## Abstract

**Background:**

Extubation assessment focuses on respiratory, neurologic, and airway readiness, whereas osmotic-metabolic status has received less attention. We examined whether pre-extubation calculated hyperosmolarity was associated with early invasive reintubation and whether this association replicated across independent critical care databases.

**Methods:**

We analyzed the first eligible extubation in each hospital admission in MIMIC-IV and independently replicated the analysis in the multicenter eICU-CRD. Calculated serum osmolality was derived from sodium, glucose, and blood urea nitrogen measured within 24 h before extubation. Modified Poisson models estimated adjusted risk ratios (RRs) for 48-h invasive reintubation comparing calculated serum osmolality >300 with ≤300 mOsm/kg, using the same prespecified adjustment set in both databases. Secondary analyses evaluated 72-h reintubation and continuous associations, with additional sensitivity analyses addressing respiratory severity, fluid and renal status, threshold stability, and competing events.

**Results:**

We included 2,639 eligible extubations in MIMIC-IV, with 317 reintubations, and 1,390 in eICU-CRD, with 93 reintubations. In MIMIC-IV, 48-h reintubation occurred in 13.6% of index extubations with calculated serum osmolality >300 mOsm/kg and 10.8% with values ≤300 mOsm/kg (adjusted RR, 1.31; 95% CI, 1.05–1.62). The association replicated in eICU-CRD, where the corresponding risks were 10.5% and 4.6% (adjusted RR, 1.88; 95% CI, 1.27–2.77). The corresponding adjusted 72-h RRs were 1.25 (95% CI, 1.02–1.52) and 1.53 (95% CI, 1.19–1.97). Additional adjustment for respiratory severity produced little change within the corresponding measured subsets, whereas the continuous association was weaker in MIMIC-IV.

**Conclusion:**

Pre-extubation calculated hyperosmolarity was associated with a higher risk of early invasive reintubation in two independent critical care databases. It may serve as an adjunctive metabolic–renal marker that complements established extubation assessment.

## Introduction

Liberation from invasive mechanical ventilation is a critical transition in intensive care. A successful spontaneous breathing trial demonstrates that a patient can sustain breathing under specified conditions, but it does not fully assess airway protection, secretion clearance, neurologic readiness, or cardiopulmonary reserve after removal of the endotracheal tube (1, 2). Early reintubation is associated with prolonged mechanical ventilation, greater intensive care exposure, and higher mortality, and both 48 and 72-h definitions are commonly used (3–5). Contemporary guidance therefore places respiratory mechanics and spontaneous breathing trial performance within a broader assessment of extubation readiness (6, 7). Preventive post-extubation respiratory support can reduce reintubation in selected high-risk patients (8, 9), while neurologic function and cough strength provide additional prognostic information (10, 11). Multivariable risk profiles and targeted support strategies have also been evaluated (12, 13). Even with these approaches, extubation failure remains difficult to anticipate from respiratory assessment alone.

Metabolic and homeostatic status immediately before extubation has received less attention. Water–solute imbalance, glycemic stress, and renal or catabolic burden may coexist with limited physiologic reserve and affect mental status, respiratory muscle performance, hemodynamic tolerance, and fluid distribution during the transition to unsupported breathing. These features are rarely considered as a unified domain in extubation assessment. This gap is particularly relevant to electronic health record research, in which routine laboratory measurements are widely available, whereas bedside measures such as cough strength, secretion burden, and rapid shallow breathing index are often incomplete or poorly harmonized.

Calculated serum osmolality (cOsm) integrates sodium, glucose, and blood urea nitrogen according to the formula 2 × sodium + glucose/18 + blood urea nitrogen/2.8. Abnormal calculated osmolality has been associated with mortality and acute kidney injury across several critically ill populations (14–18). cOsm is not interchangeable with directly measured serum osmolality (19) or with effective osmolality, which excludes urea (20). It therefore provides a pragmatic biochemical summary of water–solute balance, glycemic stress, and renal or catabolic burden. A recent MIMIC-IV study reported a U-shaped association between serum osmolality and mortality in patients with acute respiratory failure (21). Whether this broader metabolic–renal state is associated with early invasive reintubation remains uncertain.

We evaluated whether pre-extubation calculated hyperosmolarity was associated with early invasive reintubation in MIMIC-IV and whether the association could be independently replicated in the multicenter eICU Collaborative Research Database (eICU-CRD). We also examined the continuous exposure–outcome relationship, competing events, and the contributions of individual cOsm components, while prognostic analyses were retained as secondary benchmarks to distinguish association replication from incremental prediction. We hypothesized that calculated hyperosmolarity would identify a higher-risk metabolic–renal phenotype associated with early invasive reintubation across independent ICU datasets.

## Materials and methods

### Study design and data sources

This retrospective dual-database cohort study used MIMIC-IV version 3.1 as the primary analytical cohort and eICU-CRD version 2.0 as an independent multicenter replication cohort. Jiaxiang Bian completed the MIMIC-IV Collaborative Institutional Training Initiative (CITI) course (Record ID: 13501033), and Youli Chen was certified to access eICU-CRD (Certification ID: 66408921). MIMIC-IV contains deidentified records from Beth Israel Deaconess Medical Center collected between 2008 and 2022, whereas eICU-CRD contains intensive care data from multiple US hospitals collected during 2014 and 2015 (22, 23). The local database extracts were obtained on January 7, 2026. Eligibility criteria, exposure windows, outcome definitions, units, and statistical estimands were specified before comparative analyses.

The two databases were processed independently. Patient-level records were not pooled, and eICU-CRD was not used to select variables, thresholds, transformations, or model parameters. MIMIC-IV defined the primary analytical specification, which was then applied unchanged to eICU-CRD. The primary objective was association analysis with independent replication; prognostic evaluation was secondary. Reporting followed the Strengthening the Reporting of Observational Studies in Epidemiology (STROBE) statement (24), with the secondary prognostic analyses additionally informed by TRIPOD+AI recommendations (25).

Both databases contain deidentified data released to credentialed users under data-use agreements. Their source publications describe the relevant institutional review approvals and waivers of individual informed consent (22, 23). Additional local institutional ethics review was not required under the authors' institutional policy because the present study involved only secondary analysis of deidentified data.

### Study population and ventilation phenotype

We included adults with an identifiable episode of invasive mechanical ventilation (IMV) followed by an eligible extubation. The first eligible extubation in each hospital admission was selected as the index event. Repeated admissions were retained in the primary analysis, with restriction to the first eligible admission per patient in sensitivity analysis. We excluded tracheostomy conversion, terminal or palliative extubation, death-related cessation of ventilation, and boundaries that could not be classified reliably.

Hospital-level IMV trajectories were reconstructed from airway and procedure records, invasive ventilator documentation, and compatible respiratory charting. Noninvasive ventilation, continuous or bilevel positive airway pressure, high-flow nasal oxygen, and Standby ventilator modes were not classified as IMV. Candidate extubations were cross-checked against transfer, discharge, death, and tracheostomy records, and subsequent invasive airway evidence was sought across all intensive care unit (ICU) stays within the same hospital admission. The primary cohort included eligible extubation events regardless of whether extubation intent was explicitly documented. Sensitivity analyses excluded events with structured unplanned-extubation labels in MIMIC-IV and reintubations accompanied by procedural evidence within ±6 h. eICU-CRD had no comparably reliable structured field for unplanned extubation. Database-specific source rules are provided in the Supplementary Methods.

### Exposure assessment

Calculated serum osmolality (cOsm) was defined as:

cOsm=2×sodium+glucose/18+bloodureanitrogen/2.8

Sodium was expressed in mmol/L and glucose and blood urea nitrogen (BUN) in mg/dL. We selected the most recent complete component set within 24 h before extubation, requiring all measurements to precede the index extubation and the maximum separation among component collection times to be no more than 2 h. Calculated hyperosmolarity was defined *a priori* as cOsm >300 mOsm/kg. Continuous analyses evaluated cOsm per 10-mOsm/kg increase, per database-specific standard deviation (SD), and using restricted cubic splines.

Albumin-corrected anion gap (ACAG) was evaluated as an exploratory marker and calculated as sodium − chloride − bicarbonate + 2.5 × (4.0 − albumin), with electrolytes expressed in mmol/L and albumin in g/dL. The electrolyte set and closest albumin measurement were obtained within 24 h before extubation. A separate 48-h albumin variable was reserved for sensitivity analyses and was not used in the reported ACAG models.

### Outcomes and competing events

The primary outcome was invasive reintubation within 48 h after index extubation; reintubation within 72 h was secondary. Noninvasive respiratory support alone did not meet the outcome definition. Outcomes were ascertained across the entire hospital admission. For the primary binary outcome, death before reintubation was classified as no observed reintubation. Death without prior reintubation was also treated as a competing event in cause-specific Cox and Fine–Gray analyses. Composite outcomes of reintubation or death were evaluated at 48 and 72 h.

### Covariates and missing data

The prespecified adjustment set comprised age, sex, ventilation duration, pre-extubation respiratory rate, oxygen saturation, Glasgow Coma Scale (GCS) score, serum creatinine, and vasopressor use during the preceding 24 h. Fixed transformations were age per 10 years centered at 65, log_2_(ventilation hours + 1), respiratory rate per 5 breaths/min centered at 20, oxygen saturation per 5 percentage points centered at 95, GCS deficit from 15 per 3 points, and log_2_ creatinine. Sex and vasopressor use were binary.

Candidate respiratory variables for a harmonized expanded adjustment set included the PaO_2_/FiO_2_ (P/F) ratio, PaCO_2_, arterial pH, positive end-expiratory pressure (PEEP), and lactate. Eligibility for this shared model required harmonization across databases, no more than 30% missingness in either cohort, recorded timing for at least 95% of observed values, and measurement within 24 h before extubation for at least 95% of timed observations. Because the P/F ratio did not meet the cross-database completeness criterion for inclusion in the harmonized primary model, it was evaluated separately in measured-subset sensitivity analyses.

Missing covariates were imputed separately within each database using chained equations with 30 imputed datasets, 20 iterations, and a prespecified seed. Predictive mean matching was used for continuous variables and logistic or multinomial regression for categorical variables, as appropriate. The outcome was included as a predictor in the imputation models for association analyses but not for prognostic analyses. Exposures, outcomes, and identifiers were not imputed. Estimates and covariance matrices were combined using Rubin's rules (26, 27); complete-case analyses were performed as sensitivity analyses.

### Association analyses

Continuous variables are reported as median (interquartile range) and categorical variables as *n* (%). Between-database differences were summarized using standardized mean differences. Observed risks and absolute risk differences were calculated according to calculated hyperosmolarity status.

Modified Poisson regression estimated adjusted risk ratios (RRs) and 95% confidence intervals (CIs) (28). MIMIC-IV used observation-level HC0 robust covariance, whereas eICU-CRD used hospital-cluster robust covariance (vcovCL, type HC0) within each imputed dataset, followed by Rubin pooling. Logistic regression provided complementary adjusted odds ratios. Threshold sensitivity analyses compared cOsm >295, >300, and >305 mOsm/kg, while four categories (<290, 290–<300, 300–<310, and ≥310 mOsm/kg) were examined descriptively.

Restricted cubic splines were fitted on the modified Poisson RR scale (29, 30). Knots were fixed at the MIMIC-IV 10th, 50th, and 90th percentiles (283.17, 297.53, and 320.51 mOsm/kg), with 300 mOsm/kg as the reference. The same knots, reference value, and transformations were applied unchanged to eICU-CRD. Overall and nonlinear Wald tests were reported separately.

Biochemical component analyses used separate models with the same adjustment set to evaluate cOsm >300 mOsm/kg, cOsm per database-specific SD, sodium per 5 mmol/L, BUN per 10 mg/dL, and glucose per 50 mg/dL. cOsm and its individual components were not entered simultaneously in the same model.

### Secondary prognostic benchmarking

The base logistic model contained the eight prespecified clinical covariates. Separate augmented models added cOsm >300 mOsm/kg, sodium per 5 mmol/L, BUN per 10 mg/dL, or glucose per 50 mg/dL. Models were developed in MIMIC-IV; the base and cOsm models underwent internal validation using 1,000 bootstrap resamples across the 30 imputed datasets (31, 32). Intercepts, coefficients, and variable transformations were then fixed and applied directly to eICU-CRD.

Performance was assessed using the area under the receiver operating characteristic curve (AUROC), area under the precision-recall curve (PR-AUC), Brier score, calibration intercept, calibration slope, and observed-to-expected ratio (33, 34). Differences from the base model were estimated using 2,000 paired bootstrap resamples of eICU hospitals. Decision-curve analysis evaluated risk thresholds from 0.01 to 0.25 (35). Original external performance was reported before intercept-only and intercept-plus-slope recalibration.

### Sensitivity and exploratory analyses

Sensitivity analyses restricted cOsm component timing to 12 h, required at least 24 h of IMV, retained only the first eligible admission per patient, and excluded identifiable unplanned extubations and potentially procedure-related reintubations. Respiratory sensitivity analyses evaluated pre-extubation P/F ratio and FiO_2_/PEEP within measured subsets, comparing the clinical and extended models in identical patients. Fluid and renal analyses incorporated recorded 24-h fluid balance, loop-diuretic exposure, and creatinine-based acute kidney injury (AKI); identifiable hypertonic therapy was excluded in a separate sensitivity analysis. Post-extubation noninvasive ventilation (NIV) and high-flow nasal oxygen (HFNO) were evaluated using a 6-h landmark analysis rather than treated as baseline covariates.

We additionally examined effective osmolality (2 × sodium + glucose/18), serial cOsm measurements, and an exploratory MIMIC-IV Youden threshold assessed using 1,000 bootstrap resamples and then applied unchanged to eICU-CRD. Death before reintubation was evaluated using cause-specific Cox models based on the 30 imputed datasets used for the primary covariates and Fine–Gray subdistribution hazard models implemented using weighted Cox regression among complete cases for those covariates. Robust covariance was clustered by patient in MIMIC-IV and by hospital in eICU-CRD. A further eICU-CRD sensitivity analysis was restricted to extubations supported by higher-tier evidence.

Albumin-corrected anion gap was retained as an exploratory supplementary marker. Because only 26 eICU-CRD events had calculable ACAG, external analyses were limited to crude models and Firth logistic regression adjusted for age, sex, and ventilation duration. Detailed definitions, timing rules, measurement coverage, and implementation are provided in the Supplementary Methods. All tests were two-sided with *α* = 0.05. Analyses were performed using R version 4.5.2.

## Results

### Cohort construction and characteristics

Phenotype reconstruction identified 2,679 MIMIC-IV and 1,580 eICU-CRD hospital admissions with an eligible index extubation. Application of the prespecified cOsm timing criteria yielded 2,639 MIMIC-IV index extubations with 317 reintubations within 48 h and 1,390 eICU-CRD index extubations with 93 events (Figure 1). The MIMIC-IV cohort comprised 2,639 unique patients. In eICU-CRD, the 1,390 index extubations represented 1,334 unique patients; 41 patients contributed at least two eligible admissions, with a maximum of four.

**Figure 1:**
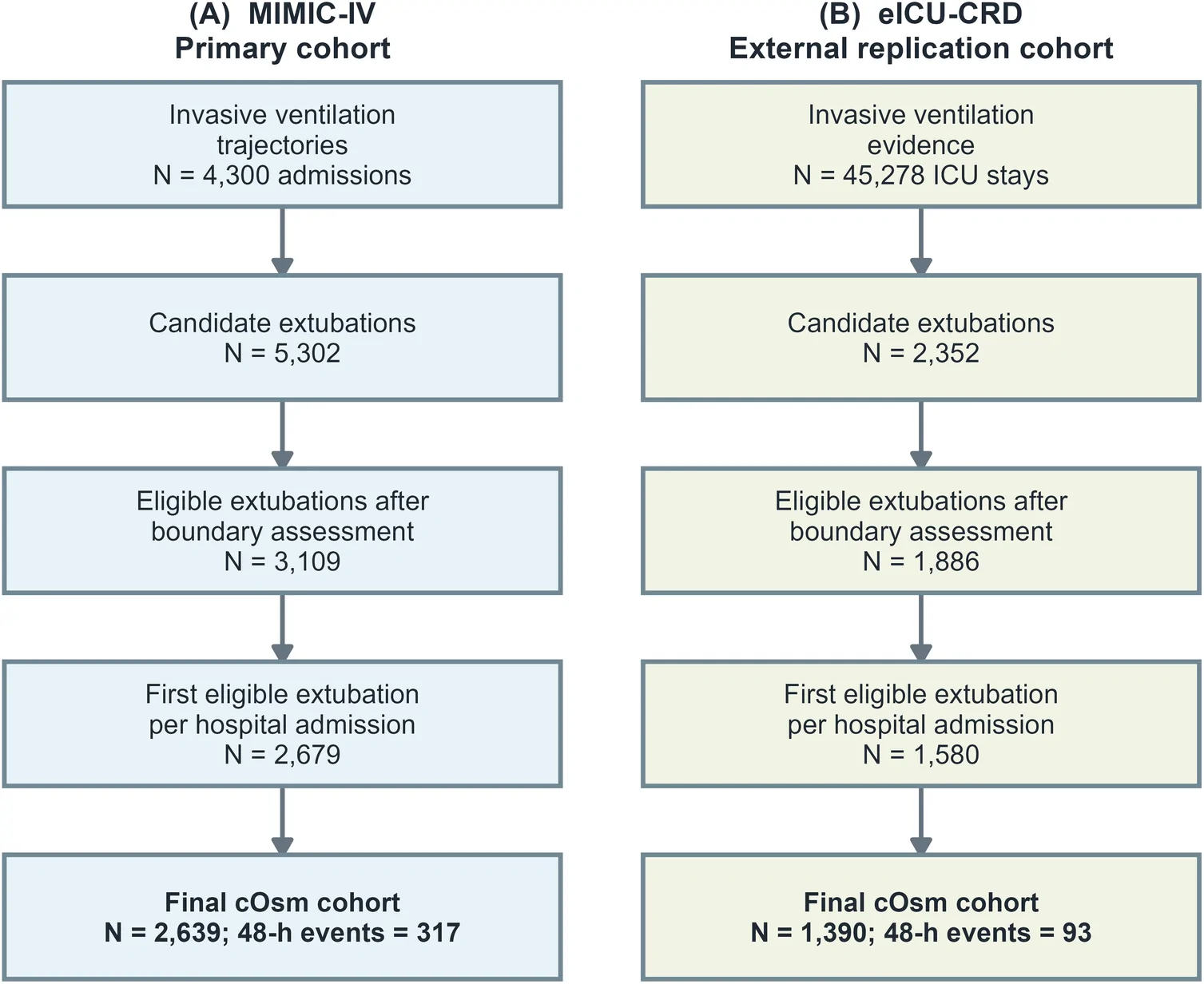
Dual-database cohort screening and phenotype reconstruction. MIMIC-IV was used as the primary analytical cohort and eICU-CRD as the independent multicenter replication cohort. Invasive ventilation trajectories and candidate extubations were reconstructed separately. The final cOsm cohorts comprised 2,639 MIMIC-IV eligible index extubations with 317 reintubations within 48 h and 1,390 eICU-CRD eligible index extubations with 93 events.

Median age was 65 years (interquartile range, 53–76) in MIMIC-IV and 63 years (53–72) in eICU-CRD. Median ventilation duration was 69.4 h (41.6–143.6) and 51.5 h (23.9–125.4), respectively. The largest standardized differences between the two cohorts were observed for Glasgow Coma Scale score (standardized mean difference, 0.87) and vasopressor use (standardized mean difference, 0.63). Median cOsm was 297.5 mOsm/kg (289.1–308.2) in MIMIC-IV and 295.2 mOsm/kg (288.2–304.4) in eICU-CRD; 42.6% and 35.0%, respectively, had cOsm >300 mOsm/kg. The median intervals from the representative component set to extubation were 8.3 h (5.3–11.2) and 8.8 h (4.9–13.1), respectively (Table 1).

**Table 1:** Cohort characteristics.

Characteristic	MIMIC-IV	eICU-CRD	SMD
Eligible index extubations, *n*	2,639	1,390	
Age, years	65.0 [53.0, 76.0]	63.0 [53.0, 72.0]	0.104
Mechanical ventilation duration, h	69.4 [41.6, 143.6]	51.5 [23.9, 125.4]	0.050
Respiratory rate, breaths/min	19.0 [15.0, 23.0]	20.0 [17.0, 24.0]	0.256
Oxygen saturation, %	98.0 [96.0, 100.0]	98.0 [95.0, 99.0]	0.211
Glasgow Coma Scale score	10.0 [9.0, 11.0]	13.0 [10.0, 15.0]	0.867
Creatinine, mg/dL	1.1 [0.7, 1.8]	1.0 [0.7, 1.6]	0.054
Creatinine-based AKI, *n*/*N* (%)	735/2,629 (28.0%)	204/1,300 (15.7%)	0.300
Sodium, mmol/L	140.0 [137.0, 143.0]	140.0 [137.0, 143.0]	0.057
Glucose, mg/dL	127.0 [104.0, 155.0]	128.0 [108.0, 152.0]	0.003
Blood urea nitrogen, mg/dL	25.0 [15.0, 43.0]	21.0 [14.0, 34.0]	0.242
Calculated serum osmolality, mOsm/kg	297.5 [289.1, 308.2]	295.2 [288.2, 304.4]	0.181
Representative cOsm measurement to extubation, h	8.3 [5.3, 11.2]	8.8 [4.9, 13.1]	0.179
Male sex	1,543 (58.5%)	823 (59.3%)	0.016
Vasopressor use within 24 h	1,263 (47.9%)	272 (19.6%)	0.627
cOsm >300 mOsm/kg	1,124 (42.6%)	486 (35.0%)	0.157
Invasive reintubation within 48 h	317 (12.0%)	93 (6.7%)	0.184
Invasive reintubation within 72 h	376 (14.2%)	119 (8.6%)	0.180

Values are median (interquartile range), *n* (%), or *n*/*N* (%) for AKI. SMDs are absolute values. AKI was defined using KDIGO-compatible serum creatinine changes based on available in-hospital comparators; urine-output and renal-replacement-therapy criteria were not included. AKI, acute kidney injury; SMD, standardized mean difference; cOsm, calculated serum osmolality.

### Primary association and independent replication

In MIMIC-IV, reintubation within 48 h occurred after 153 of 1,124 extubations (13.6%) with cOsm >300 mOsm/kg and 164 of 1,515 extubations (10.8%) with cOsm ≤300 mOsm/kg. The absolute risk difference was 2.8 percentage points, and the adjusted RR was 1.31 (95% CI, 1.05–1.62) (Figures 2, 3; Table 2).

**Figure 2:**
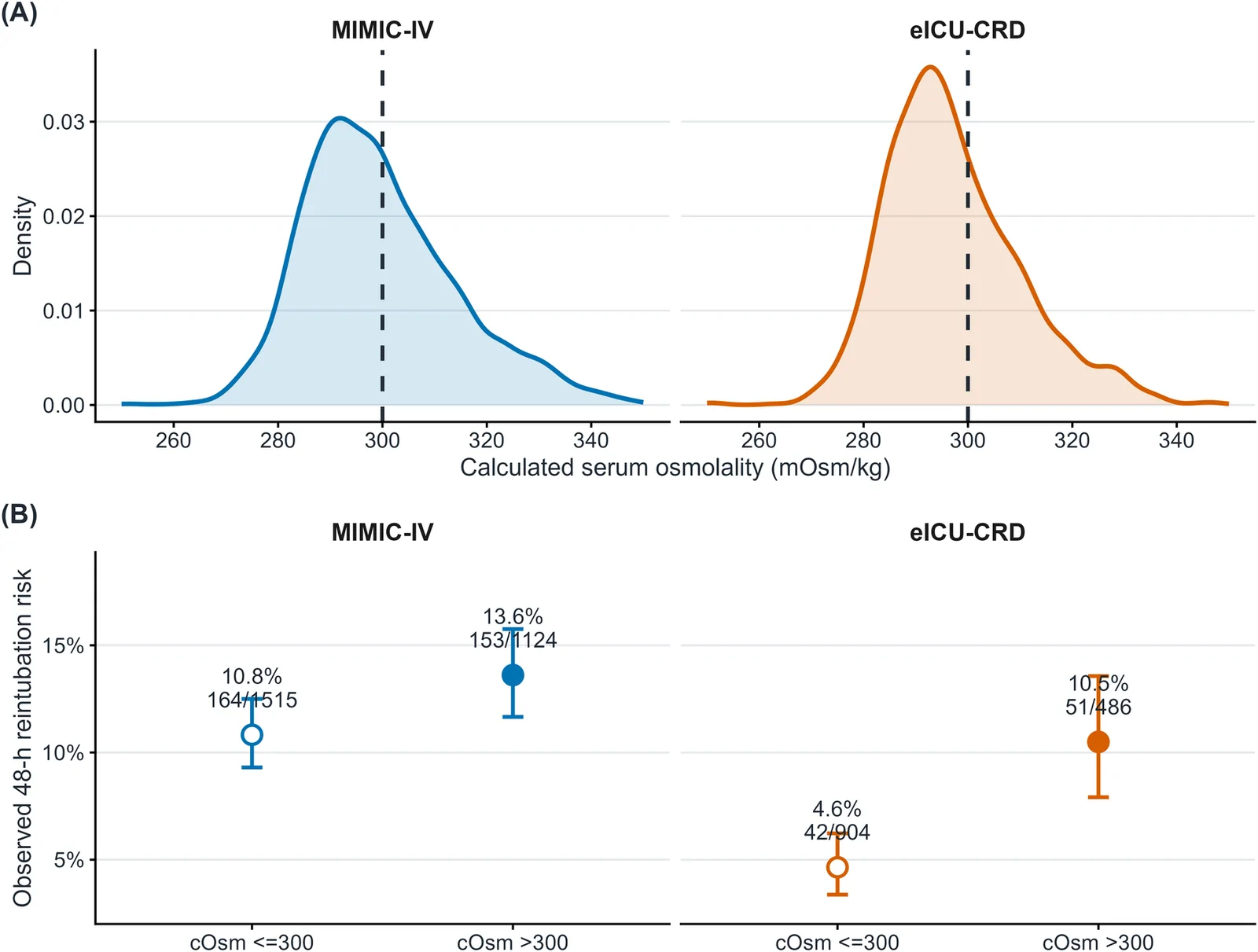
Pre-extubation calculated serum osmolality distributions and observed 48-h reintubation risk. Panel **(A)** shows cOsm distributions in MIMIC-IV and eICU-CRD; the dashed vertical line denotes 300 mOsm/kg. Panel **(B)** shows observed risks for cOsm ≤300 and >300 mOsm/kg. Points are observed risks, bars are exact binomial 95% confidence intervals, and labels give events and denominators.

**Figure 3:**
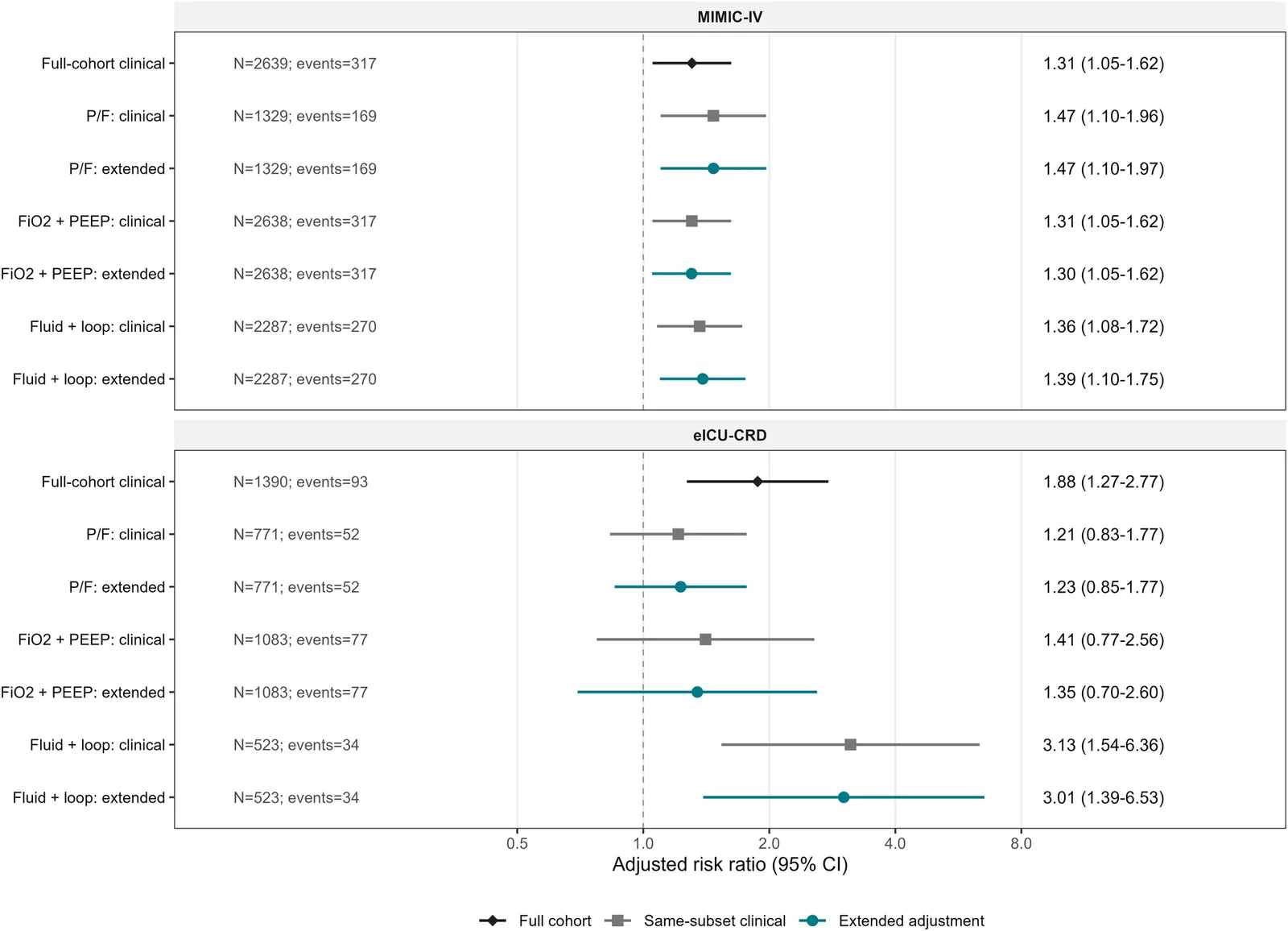
Primary and same-subset associations of calculated hyperosmolarity with 48-h invasive reintubation. The full-cohort estimate compares cOsm >300 with ≤300 mOsm/kg. Paired estimates compare the clinical model and extended models incorporating P/F ratio, FiO_2_/PEEP, or fluid balance and loop-diuretic exposure in identical measured subsets. Points show adjusted RRs and bars show 95% confidence intervals; labels report events and sample sizes. Estimates were obtained separately in each database. The eICU-CRD fluid subset contains 34 events (Supplementary Tables S15, S16).

**Table 2:** Primary associations and component benchmarking.

Marker	Outcome window	MIMIC-IV events/*N*	MIMIC-IV adjusted RR (95% CI)	eICU-CRD events/*N*	eICU-CRD adjusted RR (95% CI)
cOsm >300 vs. ≤300 mOsm/kg	48 h	317/2,639	1.31 (1.05–1.62)	93/1,390	1.88 (1.27–2.77)
cOsm >300 vs. ≤300 mOsm/kg	72 h	376/2,639	1.25 (1.02–1.52)	119/1,390	1.53 (1.19–1.97)
Calculated serum osmolality (per database-specific SD)	48 h	317/2,639	1.05 (0.94–1.17)	93/1,390	1.28 (1.07–1.53)
Sodium (per 5 mmol/L)	48 h	317/2,639	1.04 (0.94–1.14)	93/1,390	1.15 (0.97–1.37)
Blood urea nitrogen (per 10 mg/dL)	48 h	317/2,639	1.04 (0.98–1.09)	93/1,390	1.15 (1.00–1.33)
Glucose (per 50 mg/dL)	48 h	317/2,639	0.93 (0.83–1.03)	93/1,390	1.12 (0.93–1.35)

Adjusted RRs were estimated by modified Poisson regression using the same eight prespecified covariates. Each component model added one biochemical marker only. cOsm was never entered with sodium, blood urea nitrogen or glucose. eICU-CRD covariance was clustered by hospital. CI, confidence interval; cOsm, calculated serum osmolality; RR, risk ratio; SD, standard deviation.

In eICU-CRD, the corresponding risks were 51 of 486 (10.5%) and 42 of 904 (4.6%), yielding an absolute risk difference of 5.8 percentage points and an adjusted RR of 1.88 (95% CI, 1.27–2.77). At 72 h, the adjusted RRs were 1.25 (95% CI, 1.02–1.52) in MIMIC-IV and 1.53 (95% CI, 1.19–1.97) in eICU-CRD (Table 2).

### Respiratory and fluid/renal sensitivity analyses

Additional adjustment for the PaO_2_/FiO_2_ (P/F) ratio produced little change within the corresponding measured subsets. In MIMIC-IV (1,329 extubations; 169 events), the adjusted RR was 1.47 (95% CI, 1.10–1.96) before and 1.47 (95% CI, 1.10–1.97) after P/F adjustment. In eICU-CRD (771 extubations; 52 events), the corresponding estimates were 1.21 (95% CI, 0.83–1.77) and 1.23 (95% CI, 0.85–1.77). The lower eICU-CRD estimate was therefore already present in the P/F-measured subset before P/F adjustment. In the higher-coverage FiO_2_/PEEP analysis, same-subset RRs changed from 1.31 to 1.30 in MIMIC-IV and from 1.41 to 1.35 in eICU-CRD (Figure 3; Supplementary Tables S14, S15). Analyses of documented NIV/HFNO use and the 6-h landmark cohorts are reported in Supplementary Table S18.

Adjustment for recorded 24-h fluid balance and loop-diuretic exposure changed the same-subset MIMIC-IV RR from 1.36 (95% CI, 1.08–1.72) to 1.39 (95% CI, 1.10–1.75). In the smaller eICU-CRD subset with 34 events, the corresponding RRs were 3.13 (95% CI, 1.54–6.36) and 3.01 (95% CI, 1.39–6.53) (Figure 3; Supplementary Table S16). Creatinine-based AKI was present in 28.0% of evaluable MIMIC-IV extubations and 15.7% of evaluable eICU-CRD extubations. Exclusion of identifiable hypertonic therapy in MIMIC-IV yielded an adjusted RR of 1.33 (95% CI, 1.07–1.66) (Supplementary Table S17).

### Exposure form and physiologic analyses

Estimates across the neighboring thresholds of >295, >300, and >305 mOsm/kg were concordant in direction, ranging from 1.07 to 1.31 in MIMIC-IV and from 1.39 to 1.88 in eICU-CRD (Table 3; Supplementary Table S2 and Supplementary Figure S1). The exploratory MIMIC-IV Youden threshold was 301.964 mOsm/kg. Applying this threshold unchanged to eICU-CRD yielded an adjusted RR of 2.04 (95% CI, 1.41–2.96). Bootstrap-derived thresholds remained centered near 300 mOsm/kg but showed appreciable dispersion (Supplementary Table S19 and Supplementary Figure S16).

**Table 3:** Targeted sensitivity and exposure-form analyses.

Analysis	MIMIC-IV *N*/events	MIMIC-IV adjusted RR (95% CI)	eICU-CRD *N*/events	eICU-CRD adjusted RR (95% CI)
cOsm >295 vs. ≤295 mOsm/kg	2,639/317	1.07 (0.86–1.32)	1,390/93	1.39 (0.90–2.15)
cOsm >300 vs. ≤300 mOsm/kg	2,639/317	1.31 (1.05–1.62)	1,390/93	1.88 (1.27–2.77)
cOsm >305 vs. ≤305 mOsm/kg	2,639/317	1.21 (0.96–1.53)	1,390/93	1.56 (1.01–2.40)
cOsm, per 10 mOsm/kg	2,639/317	1.03 (0.96–1.11)	1,390/93	1.21 (1.06–1.39)
Effective osmolality, per 10 mOsm/kg	2,639/317	1.02 (0.93–1.11)	1,390/93	1.18 (0.96–1.45)
Derived cOsm threshold >301.964 mOsm/kg	Derivation cohort	Not reported (derivation)	1,390/93	2.04 (1.41–2.96)
Reintubation or death within 48 h	2,639/684	1.09 (0.97–1.23)	1,389/133	1.42 (1.13–1.78)

RRs were estimated using the eight prespecified clinical covariates. Exposure-form analyses use the full primary cohorts. The MIMIC-derived threshold is exploratory and was applied unchanged to eICU-CRD. Threshold derivation and bootstrap stability are detailed in Supplementary Table S19. Effective osmolality excludes BUN. The 48-h composite includes 2,639 MIMIC-IV admissions/684 events and 1,389 eICU-CRD admissions/133 events; one eICU-CRD composite outcome was unavailable. Detailed competing-risk and paired respiratory/fluid models are in Supplementary Tables S5, S15, S16. CI, confidence interval; RR, risk ratio.

For each 10-mOsm/kg increase in cOsm, the adjusted RR was 1.03 (95% CI, 0.96–1.11; *P* = 0.394) in MIMIC-IV and 1.21 (95% CI, 1.06–1.39; *P* = 0.006) in eICU-CRD. Per database-specific SD, the corresponding estimates were 1.05 (95% CI, 0.94–1.17) and 1.28 (95% CI, 1.07–1.53). Restricted cubic spline analyses showed no evidence of nonlinearity in either cohort (MIMIC-IV, *P* = 0.800; eICU-CRD, *P* = 0.377). Overall spline *P* values were 0.693 in MIMIC-IV and <0.001 in eICU-CRD (Figure 4; Supplementary Table S3).

**Figure 4:**
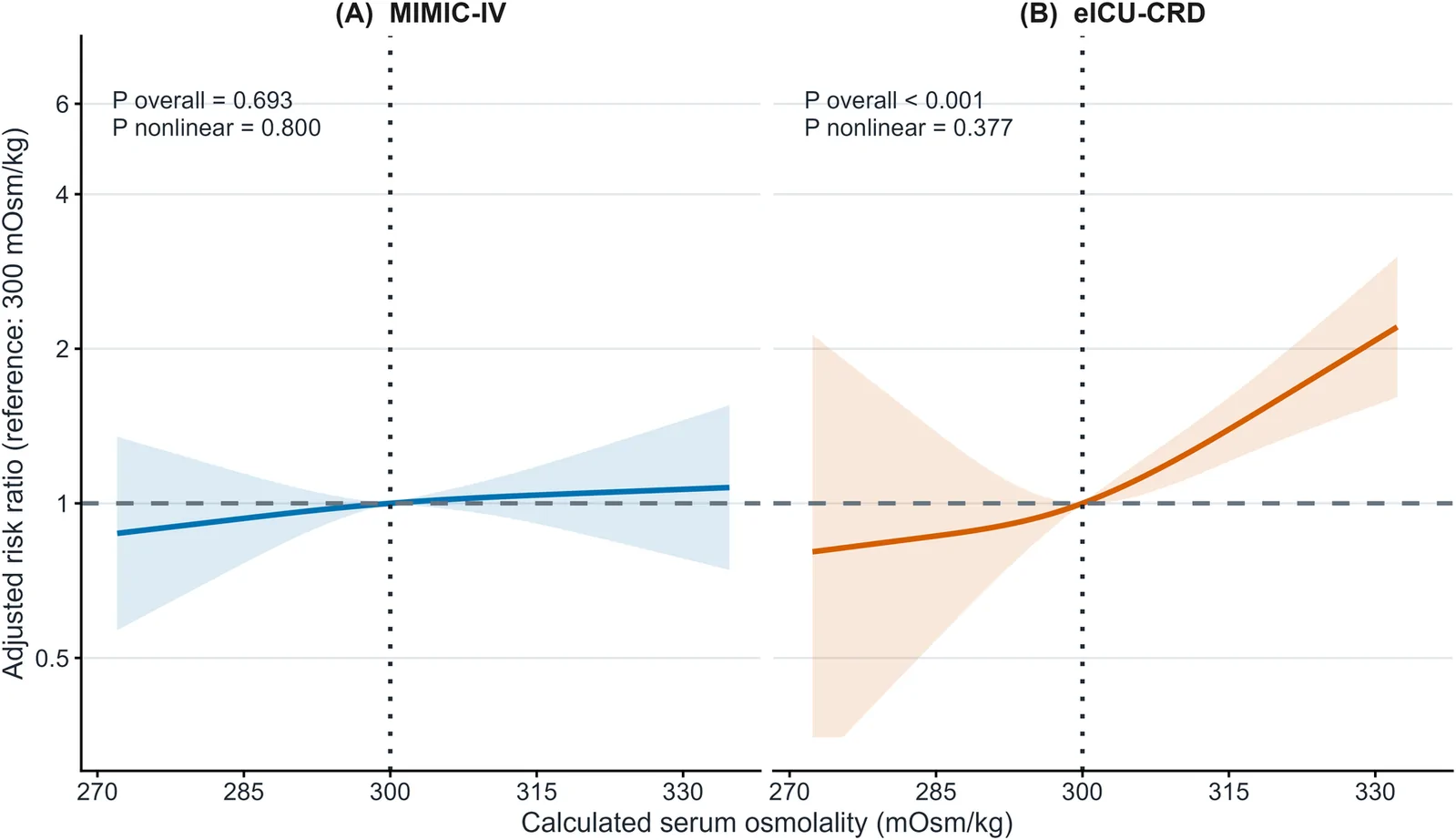
Continuous association between pre-extubation calculated serum osmolality and 48-h invasive reintubation. Restricted cubic spline estimates are shown for MIMIC-IV and eICU-CRD with 300 mOsm/kg as the reference. Solid lines show adjusted RRs and shaded bands show 95% confidence intervals. Knots at 283.17, 297.53 and 320.51 mOsm/kg were determined in MIMIC-IV and applied unchanged to eICU-CRD. Overall and nonlinear Wald tests are displayed. Panel (A) shows MIMIC-IV, and panel (B) shows eICU-CRD.

The individual biochemical components did not show the same cross-database association pattern as calculated hyperosmolarity. None of the individual component estimates had 95% CIs excluding 1.00 in both databases (Table 2; Supplementary Tables S11 and S13).

For effective osmolality, adjusted RRs per 10-mOsm/kg increase were 1.02 (95% CI, 0.93–1.11) in MIMIC-IV and 1.18 (95% CI, 0.96–1.45) in eICU-CRD (Table 3; Supplementary Table S20 and Supplementary Figure S17). These estimates were weaker than those observed for cOsm.

Pre-extubation changes in cOsm were modest and substantially overlapped between patients with and without reintubation. Among initially hyperosmolar patients with available post-extubation measurements, early normalization was more frequent in those without reintubation, although measurement opportunity differed by outcome (Supplementary Table S21 and Supplementary Figure S14). The exploratory albumin-corrected anion gap analysis did not show a corresponding independent association (Supplementary Table S10).

### Competing-event and phenotype analyses

Within 48 h after extubation, death without prior reintubation occurred in 367 MIMIC-IV and 40 eICU-CRD admissions. The cause-specific hazard ratios were 1.29 (95% CI, 1.02–1.63) in MIMIC-IV and 1.96 (95% CI, 1.34–2.89) in eICU-CRD. The corresponding Fine–Gray subdistribution hazard ratios were 1.34 (95% CI, 1.06–1.69) and 2.03 (95% CI, 1.45–2.86), respectively (Supplementary Table S5 and Supplementary Figures S2 and S15). For the 48-h composite outcome of reintubation or death, the adjusted RRs were 1.09 (95% CI, 0.97–1.23) in MIMIC-IV and 1.42 (95% CI, 1.13–1.78) in eICU-CRD, with greater attenuation in MIMIC-IV (Table 3).

In MIMIC-IV, 40 primary cOsm index extubations were linked to explicit unplanned-extubation labels; excluding these events yielded an adjusted RR of 1.30 (95% CI, 1.04–1.64). No comparably reliable explicit unplanned-extubation field was identified in eICU-CRD. Source-record review within a fixed ±6-h window identified 37 of 317 MIMIC-IV 48-h reintubations (11.7%) and 5 of 93 eICU-CRD reintubations (5.4%) as potentially procedure-related. Excluding all admissions flagged at either 48 or 72 h yielded adjusted RRs of 1.32 (95% CI, 1.04–1.66) and 1.90 (95% CI, 1.25–2.89), respectively.

Restriction to the first eligible admission per patient yielded an eICU-CRD RR of 1.89 (95% CI, 1.24–2.88). Requiring all cOsm components to be measured within 12 h before extubation yielded RRs of 1.49 (95% CI, 1.17–1.90) in MIMIC-IV and 2.06 (95% CI, 1.43–2.96) in eICU-CRD (Supplementary Tables S6–S8). Restricting eICU-CRD to Tier 1/2 extubation evidence retained 835 admissions and 65 events and yielded an adjusted RR of 1.74 (95% CI, 1.29–2.35) (Supplementary Table S7).

### Incremental prognostic information

In MIMIC-IV, apparent AUROCs were 0.5976 for the base model, 0.6093 with cOsm, 0.5988 with sodium, 0.6008 with blood urea nitrogen, and 0.5998 with glucose. For the base and cOsm models, optimism-corrected AUROCs were 0.5785 and 0.5891, respectively, and optimism-corrected Brier scores were 0.10521 and 0.10507. When the fixed MIMIC-IV coefficients were applied to eICU-CRD, the corresponding AUROCs for the five models were 0.5875, 0.6074, 0.5928, 0.5959, and 0.5827. The cOsm model showed the largest numerical increase in AUROC over the base model (0.0199; 95% CI, −0.0327 to 0.0434), although the 95% CI included zero. AUROC differences were 0.0053 (95% CI, −0.0138 to 0.0125) for sodium, 0.0084 (95% CI, −0.0101 to 0.0305) for blood urea nitrogen, and −0.0047 (95% CI, −0.0170 to 0.0072) for glucose.

External PR-AUC values were 0.1051 for the base model, 0.1229 with cOsm, 0.1086 with sodium, 0.1072 with blood urea nitrogen, and 0.1014 with glucose. The corresponding Brier scores were 0.06932, 0.06836, 0.06913, 0.06885, and 0.06942. All absolute changes in Brier score were ≤0.0010. The blood urea nitrogen model had a Brier-score difference of −0.00047 (95% CI, −0.00099 to −0.00007), whereas the cOsm difference was −0.00096 (95% CI, −0.00131 to 0.00020). Overall, incremental prognostic differences between cOsm and its individual components were small (Supplementary Table S9).

Original external validation showed overprediction of absolute risk in eICU-CRD, consistent with a calibration-in-the-large difference between the two cohorts. Calibration intercepts were −0.91 and −0.89, calibration slopes were 0.93 and 1.14, and observed-to-expected ratios were 0.45 and 0.45 for the base and cOsm models, respectively (Supplementary Figure S9). Intercept-only recalibration reduced the cOsm-model Brier score to 0.06151, while intercept-plus-slope recalibration yielded a Brier score of 0.06145. Recalibrated estimates were reported separately from the original external validation results.

## Discussion

Pre-extubation cOsm >300 mOsm/kg was associated with a higher risk of invasive reintubation within 48 h in MIMIC-IV, and this association was independently replicated in the multicenter eICU-CRD. The 72-h endpoint, competing-risk analyses, and airway-phenotype sensitivity analyses were directionally consistent with the primary finding. Continuous associations differed between databases, although neither spline analysis showed evidence of nonlinearity. No individual biochemical component reproduced the categorical cOsm association across both cohorts. Incremental prognostic gains were modest, distinguishing the replicated association from the marker's limited contribution to prediction. Targeted respiratory and fluid/renal analyses further refined this interpretation, with little change after additional adjustment within the corresponding measured subsets.

Extubation decisions are primarily informed by respiratory mechanics, neurologic readiness, airway protection, cough strength, and secretion management (1, 6, 7, 10, 11). These domains remain central to extubation assessment. Additional adjustment for the P/F ratio and FiO_2_/PEEP produced little change within the corresponding measured subsets, indicating that the measured respiratory variables did not account for the observed association within those subsets.

The present study adds a metabolic perspective on the patient's physiologic state immediately before extubation. Sodium reflects water–solute balance, glucose captures part of the acute metabolic response, and blood urea nitrogen reflects both renal handling and catabolic burden (16, 18, 20). Their combination may capture a metabolic–renal state that is not represented by any single component model. Such an integrated signal is biologically plausible during the transition from invasive ventilation, when the work of breathing increases and positive-pressure support is withdrawn.

Previous critical care studies have linked abnormal calculated osmolality with mortality and acute kidney injury (14–18), and more recent work in acute respiratory failure has reported an association with short-term mortality (21). Our findings extend this literature to a time-specific outcome: invasive reintubation after extubation. cOsm was assessed close to extubation rather than at ICU admission, aligning the exposure with the physiologic state immediately preceding liberation from mechanical ventilation. Its replication across two independent databases suggests that this metabolic–renal domain may also be relevant to extubation outcome, rather than being confined to broader mortality or organ-dysfunction endpoints.

Component analyses further refine this interpretation. Sodium, blood urea nitrogen, and glucose were evaluated separately on prespecified clinical scales using the same covariate adjustment. None showed 95% CIs excluding 1.00 in both databases, whereas calculated hyperosmolarity showed a categorical association in each cohort. The fluid-balance, loop-diuretic, and creatinine-based AKI analyses were also compatible with a broader metabolic–renal interpretation. Associations with effective osmolality were weaker, suggesting that effective tonicity alone does not account for the full cOsm signal. Nevertheless, cOsm remains a calculated summary rather than a direct measurement of serum osmolality (19). Its potential value lies in integrating routinely available biochemical information at a clinically relevant time point.

The threshold analyses help clarify how the 300-mOsm/kg value should be interpreted. Estimates at >295, >300, and >305 mOsm/kg were directionally concordant, although their magnitude and precision varied. The exploratory MIMIC-IV threshold of 301.964 mOsm/kg was close to the prespecified 300-mOsm/kg value and remained associated with reintubation when applied unchanged to eICU-CRD, although bootstrap-derived thresholds showed substantial dispersion. The continuous association was stronger in eICU-CRD than in MIMIC-IV. These findings support 300 mOsm/kg as a pragmatic stratification point rather than a sharply defined biological or treatment threshold. The greater attenuation of the MIMIC-IV estimate for the reintubation-or-death composite further illustrates that the magnitude of association depends partly on the clinical endpoint being modeled.

The prognostic analyses addressed a different question. Adding calculated hyperosmolarity produced the largest numerical increase in external AUROC among the biochemical markers examined, but the confidence interval included zero. Differences in PR-AUC and Brier score were also small. The two cohorts also differed in reference-group reintubation risk (10.8% vs. 4.6%), GCS, vasopressor use, and ventilation duration. Differences in case mix, extubation practice, and source-record ascertainment may have contributed to these contrasts. Fixed MIMIC-IV predictions overestimated absolute risk in eICU-CRD, with observed-to-expected ratios of approximately 0.45. Thus, a marker may retain a reproducible relative association while adding little to transportable absolute-risk prediction. cOsm is therefore better interpreted as an additional risk signal within a broader extubation assessment than as a replacement for respiratory, airway, or neurologic evaluation.

The study has several strengths. The two databases were analyzed independently using common definitions, the airway phenotype was reconstructed across the entire hospital admission, and the eICU-CRD analyses accounted for hospital-level clustering. Exposure timing, variable transformations, covariates, and spline knots were fixed before external replication. Component analyses used the same adjustment set with one biochemical marker entered at a time, and prognostic coefficients were fixed before application to eICU-CRD. Additional respiratory, fluid/renal, competing-risk, and phenotype analyses, together with threshold derivation and unchanged external application, allowed the primary association to be examined across several clinically relevant analytical conditions.

The study also has limitations inherent to retrospective electronic health record data. Residual confounding remains possible, particularly because cough strength, secretion burden, and rapid shallow breathing index could not be harmonized across databases. Planned and unplanned extubation could not be distinguished reliably in eICU-CRD, and classification of potentially procedure-related reintubation depended on available source documentation. P/F ratio and fluid measurements were available only in selected subsets, while treatment documentation was incomplete, particularly for medication names and HFNO in eICU-CRD. AKI was defined from serum creatinine rather than complete urine-output and kidney-replacement-therapy criteria. Explicit causes of reintubation were documented for too few events to support cause-specific comparisons, and post-extubation laboratory sampling differed according to measurement opportunity. cOsm was calculated rather than directly measured. In addition, the eICU-CRD primary analysis included 93 reintubation events, with substantially fewer events in treatment and fluid-related subgroup analyses. Finally, both databases reflect US critical care practice, and replication in contemporary non-US cohorts would strengthen generalizability.

## Conclusion

Pre-extubation calculated hyperosmolarity was associated with a higher risk of early invasive reintubation in MIMIC-IV, and this association was independently replicated in the multicenter eICU-CRD. Additional analyses were consistent with cOsm reflecting a broader metabolic–renal signal rather than effective tonicity alone. Because cOsm can be derived from routinely available laboratory data, it merits prospective evaluation as a complementary marker within extubation assessment.

## Data Availability

MIMIC-IV version 3.1 and eICU-CRD version 2.0 are available to credentialed users through PhysioNet under their respective data-use agreements. The databases cannot be redistributed by the authors.
