# Supplementary material for: Pre-extubation calculated serum osmolality and early invasive reintubation in critically ill adults: a dual-database study with multicenter external replication
Source: Front Med (Lausanne). 2026 Oct 21;13:1984164. doi: 10.3389/fmed.2026.1984164 (PMC13731255; doi:10.3389/fmed.2026.1984164)
Supplement: Supplementary file 1 [file Supplementaryfile1.docx]

**Supplementary Material**

**Supplementary Methods**

**Independent cohort analyses**

MIMIC-IV version 3.1 and eICU-CRD version 2.0 were processed independently using harmonized definitions. The primary cohorts, adjustment set and imputation settings were retained for all additional analyses. Each additional-variable model was compared with the clinical model in the identical observed subset.

**Ventilation and airway evidence hierarchy**

The source databases use different representations of ventilation and airway transitions. We fixed harmonized clinical definitions while retaining database-specific extraction rules. Direct invasive airway or procedure records and explicit invasive ventilator documentation were assigned the highest evidential priority. Derived invasive ventilation intervals supported by compatible respiratory charting formed the next tier. Isolated or conflicting records without a credible invasive airway context were insufficient to define extubation or reintubation.

Event sequences were reconstructed across each hospital admission. Bilevel positive airway pressure, continuous positive airway pressure, other noninvasive ventilation and high-flow nasal oxygen were distinguished from invasive support. Ventilator mode documented as Standby was not considered invasive ventilation. Candidate ventilation-end events were checked against nearby airway evidence and against tracheostomy, death, discharge and transfer boundaries. Records compatible with tracheostomy conversion, terminal or palliative extubation, death-related ventilation cessation or a temporary documentation interruption were excluded. The first eligible extubation in each hospital admission was selected.

Reintubation required renewed invasive-airway evidence after the index extubation. Outcome surveillance extended across all ICU stays in the same hospital admission, so ICU transfer did not end follow-up. Four MIMIC-IV 48-h reintubations occurred in a later ICU stay and would have been missed by index-stay-only surveillance. Noninvasive respiratory support alone did not meet the outcome definition.

**Planned, unplanned and procedure-related events**

MIMIC-IV procedureevents records contained explicit labels for patient-initiated and non-patient-initiated unplanned extubation. Among 5,302 candidate extubations, 107 source events carried an explicit unplanned label. Forty unique labels matched a primary cOsm index extubation within 5 min. These events were retained in the original phenotype because clinical intent was not part of the fixed eligibility definition, then excluded in a sensitivity analysis.

The eICU-CRD respiratory charting and treatment source values were searched for self-extubation, accidental extubation and unplanned extubation terminology. No reliable structured field or consistently populated explicit label was identified. Planned versus unplanned extubation could therefore not be perfectly distinguished in eICU-CRD.

Potential procedure-related reintubation was defined before effect estimation using a fixed ±6-h window around the renewed invasive airway event. MIMIC-IV evidence comprised transfer to a post-anesthesia care unit or ICU records for bronchoscopy, endoscopy or a surgical procedure. eICU-CRD evidence required operating-room or post-anesthesia unit admission or a treatment leaf explicitly identifying surgery, endoscopy, bronchoscopy or a related invasive procedure. Broad routine treatment labels were not sufficient. The exclusion sensitivity analysis removed admissions flagged within either 48 or 72 h.

**Laboratory alignment and exposure timing**

Sodium, glucose and blood urea nitrogen were selected from the 24 h preceding extubation. Every component preceded extubation and the maximum difference between component times was 2 h. The most recent valid set was selected. In both final cohorts, all three retained component times were identical, producing a component spread of 0 min. cOsm was calculated as 2 × sodium + glucose/18 + blood urea nitrogen/2.8, with sodium in mmol/L and glucose and blood urea nitrogen in mg/dL.

The representative time was the latest component time in MIMIC-IV and the common/mean component time in eICU-CRD. Time from this measurement to extubation was summarized using the median, interquartile range, 5th percentile and 95th percentile. Timing sensitivities required the complete component set within 12 h or 6 h. The 12-h analysis was treated as the principal timing sensitivity because it retained more observations.

The cOsm >300-mOsm/kg threshold was clinically anchored before formal modeling. The primary threshold was not selected from outcome data. An exploratory Youden analysis was performed separately as described below. Threshold sensitivity analyses compared >295, >300 and >305 mOsm/kg with the corresponding lower values using the full adjustment set. Categories of <290, 290-<300, 300-<310 and ≥310 mOsm/kg were used only for descriptive assessment, with 290-<300 mOsm/kg as the reference.

**Missing data for association analyses**

The primary cOsm cohorts were defined before imputation. Missing covariates were imputed separately in each database by chained equations with 30 imputations, 20 iterations and a reproducible master seed of 20260823 with database-specific offsets. Predictive mean matching was used for continuous covariates; logistic or multinomial logistic regression was used for categorical covariates where applicable. The observed 48-h outcome was included as a predictor of missing covariates but was not imputed. cOsm, calculated hyperosmolarity, ACAG, outcomes, patient identifiers and hospital identifiers were not imputed. Modified Poisson coefficients and robust covariance matrices were combined using Rubin’s rules.

**Missing data for prognostic analyses**

Prognostic models used independently generated imputed datasets with the same number of imputations, iterations and variable-specific methods. Outcomes were not imputed and were excluded from the predictor matrix because the intended application uses information available before extubation. The complete association and prognostic predictor matrices are included in the analysis archive.

**Primary, threshold and continuous association models**

Adjusted risk ratios were estimated with modified Poisson regression and a log link. MIMIC-IV used observation-level HC0 covariance. In eICU-CRD, each imputed dataset used hospital-cluster robust covariance from sandwich::vcovCL(type = "HC0"), followed by Rubin pooling of coefficients and covariance matrices. The primary eICU-CRD RR confidence interval was obtained from this pooled covariance. Hospital-cluster bootstrap was used separately for risk differences and external prediction-performance differences. The adjustment set comprised age per 10 years centered at 65, male sex, log2(mechanical ventilation hours + 1), respiratory rate per 5 breaths/min centered at 20, oxygen saturation per 5 percentage points centered at 95, Glasgow Coma Scale deficit from 15 per three points, log2 serum creatinine and vasopressor exposure in the preceding 24 h. No univariable screening or stepwise selection was used.

The same model was refitted for each neighboring threshold. Continuous estimates were reported per 10 mOsm/kg and per database-specific standard deviation. The MIMIC-IV standard deviation was 14.97 mOsm/kg and the eICU-CRD standard deviation was 12.87 mOsm/kg.

**Restricted cubic splines**

Restricted cubic splines were implemented within modified Poisson models so that the curve used the same RR scale as the primary analysis. Knots were fixed at the MIMIC-IV 10th, 50th and 90th percentiles: 283.17, 297.53 and 320.51 mOsm/kg. The reference was 300 mOsm/kg. The knot locations and all covariate transformations were applied unchanged to eICU-CRD. Overall and nonlinear Wald tests were calculated separately. Curves were limited to the range with adequate support and displayed on a logarithmic RR axis.

**Death as a competing event**

Time zero was the index extubation. Reintubation was event 1 and death before reintubation was event 2. Patients alive without reintubation were administratively censored at 48 or 72 h. Death status was established from hospital death/discharge records; eICU-CRD hospital discharge time was counted as a death only when hospital discharge status was expired.

Cause-specific Cox models censored at death and pooled the original 30 completed core-covariate datasets. Fine-Gray subdistribution models used weighted Cox regression in original-core complete cases [S3]. Both analyses used covariance clustered by patient in MIMIC-IV and by hospital in eICU-CRD. Cumulative incidence functions treated death as a competing event. The original binary estimand and reintubation-or-death composites were retained as distinct analyses; one unavailable eICU-CRD composite outcome was not imputed.

**Repeated patients**

Patient identifiers were used to count eligible admissions per person. The primary cohort retained the first eligible extubation within each hospital admission. A sensitivity analysis retained only the chronologically first eligible hospital admission represented in the cOsm analytical cohort for each patient. Patient-clustered covariance was additionally calculated for MIMIC-IV, although no repeated patients were present in its primary cohort.

**Prognostic models and validation**

Model 0 contained the eight prespecified harmonized clinical covariates; Model 1 added cOsm >300 mOsm/kg. Both logistic models were estimated in MIMIC-IV. Internal validation followed an impute-then-bootstrap strategy with 1,000 resamples across 30 imputed datasets, producing 30,000 valid completed-data fits per model. Optimism was estimated for AUROC, Brier score, calibration intercept and calibration slope.

The MIMIC-IV intercepts, coefficients and transformations were fixed before application to eICU-CRD. Original external performance was calculated without eICU-CRD variable selection or coefficient re-estimation. Paired 95% confidence intervals for differences in AUROC, PR-AUC and Brier score used 2,000 bootstrap resamples of hospitals with replacement, retaining all observations within each sampled hospital.

Calibration intercept was estimated with the original prediction logit as an offset, and calibration slope by regressing outcome on that logit. Intercept-only updating corrected calibration-in-the-large. A second update estimated an intercept and one common slope without re-estimating individual predictor coefficients. Updated performance did not replace the original fixed-coefficient results. Decision curves compared Models 0 and 1 with treat-all and treat-none strategies over thresholds from 0.01 to 0.25.

**Exploratory ACAG and component analyses**

ACAG was calculated as sodium - chloride - bicarbonate + 2.5 × (4.0 - albumin). Electrolytes were expressed in mmol/L and albumin in g/dL. The electrolyte set and closest albumin measurement were restricted to the 24 h before extubation. The analysis-ready files retained a separate variable using albumin from up to 48 h, but the reported ACAG models used acag_primary_24h. ACAG was analyzed per 5 mmol/L and per standard deviation. MIMIC-IV analyses included crude and fully adjusted modified Poisson and logistic models. Because the eICU-CRD ACAG subset contained only 26 events, it was limited to crude models and a Firth logistic model adjusted for age, sex and log2 ventilation duration. Spearman correlation and variance inflation factors assessed the relation between cOsm and ACAG. Exploratory component models examined sodium, glucose and blood urea nitrogen separately per standard deviation.

**Event definitions and source implementation**

The index event was the first eligible extubation within hadm_id in MIMIC-IV or patienthealthsystemstayid in eICU-CRD. ICU offsets were mapped to hospital time, and eligible invasive ventilation lasted at least 6 h. In MIMIC-IV, candidates were explicit procedure extubations or raw-corroborated ventilation ends with invasive evidence in the preceding 6 h. Tracheostomy at or before candidate +2 h, terminal-care records within 24 h, and nonexplicit transfer/death-related cessation were flagged. All 2,639 primary MIMIC-IV extubations had explicit high-confidence evidence. Nonexplicit renewed invasive evidence required more than 60 min after extubation; explicit intubation could occur sooner.

In eICU-CRD, Tier 1 required a ventilation end with preceding endotracheal evidence. Tier 2 required at least two sources, an airway transition, or ventilator-off evidence with preceding endotracheal evidence. Tier 3 used ventilator-off documentation with at least 6 h of ventilation but without stronger evidence. Conflicting simultaneous airway states, tracheostomy conversion within ±2 h, terminal-care records within ±12 h, near-death cessation and unclear discharge boundaries were excluded using structured flags. The original strict flag identified only one primary admission; the separate Tier 1/2 sensitivity retained 835. These are source-evidence categories, not manual adjudication. eICU-CRD hospital-discharge time was used as death time when discharge status was expired. Explicit reintubation indications were sought within ±1 h; diagnoses or physiology were not substituted for an indication (Supplementary Table S22 and Supplementary Figure S13).

**Respiratory measurements and additional adjustment**

MIMIC-IV arterial PaO2 used laboratory item 50821 with arterial specimen marker 52033 (ART.) or arterial chart item 220224; PaCO2 used 50818 or arterial chart item 220235. FiO2 used 223835, PEEP 220339 and pressure support 224701. eICU-CRD used corresponding laboratory and respiratory-chart labels. FiO2 percentages were converted to fractions, without inferring FiO2 from oxygen flow. Valid ranges were FiO2 0.21-1, PEEP 0-30 cm H2O and PaO2 20-700 mmHg. Each arterial PaO2 was paired with the nearest FiO2 within 1 h, both within 24 h before extubation; the latest valid pair was selected (Supplementary Table S14).

Additional respiratory and fluid/renal variables were analyzed as observed, without new imputation. Each extended model reused the original completed core covariates and was compared with the clinical model in the same admissions. Measurement coverage and differences between included and excluded patients are reported in Supplementary Table S15.

**Fluid balance, treatment exposure and creatinine-based AKI**

Timed 24-h intake and output were reconstructed before extubation. MIMIC-IV input intervals were prorated by overlap with the window, excluding rewritten records, and timed output volumes were summed. eICU-CRD used unique row-level leaf volumes rather than cumulative subtotals. Net balance required at least 18 h of input and output observation. Ambiguous aggregates, irrigation and dialysis-affected balances were left unavailable. Urine volume was summarized separately. Reliable cumulative balance over the entire ICU stay was unavailable.

Loop exposure included furosemide, bumetanide, torsemide and recognized brand names. MIMIC-IV used input records and positive electronic medication-administration records; eICU-CRD used noncancelled active orders or positive infusion records. The latter identifies documented exposure rather than verified administration. Hypertonic-saline and mannitol names were searched in pre-extubation records.

Creatinine-based AKI used the latest pre-extubation creatinine and a rise of at least 0.3 mg/dL within 48 h or at least 1.5 times the prior measured in-hospital minimum within 7 days [S1]. Missing comparators remained unavailable. Urine-output staging, outpatient reference creatinine and renal-replacement staging were not inferred. The AKI model replaced isolated creatinine while retaining fluid balance and loop exposure.

**Post-extubation respiratory support**

NIV/HFNO was identified from explicit devices, masks, treatment labels or compatible settings in 0-2, 0-6 and 6-24 h windows before reintubation. Invasive CPAP or pressure-support modes alone did not count. The 6-h landmark analysis included patients alive and reintubation-free at 6 h and assessed subsequent reintubation through 48 h separately by documented support. Absence of a support record was classified as no documented support rather than confirmed nonuse.

**Exploratory threshold and effective osmolality**

Youden index maximization used MIMIC-IV only, with higher cOsm fixed as the risk direction [S2]. One thousand admission-level bootstrap resamples described threshold dispersion. The exact threshold of 301.964285714286 mOsm/kg was then applied unchanged to eICU-CRD. This exploratory analysis did not replace the prespecified >300-mOsm/kg comparison. Effective osmolality was 2 × sodium + glucose/18, excluding BUN, and was analyzed per 10 mOsm/kg. Its modified-Poisson splines used common knots at 275.278, 287.444 and 302.178 mOsm/kg and a common reference of 287.444 mOsm/kg, the MIMIC-IV median. Overall and nonlinear Wald tests were evaluated separately.

**Serial cOsm**

Serial windows were [-72,-48), [-48,-24), [-24,-12), [-12,0), [0,24) and [24,48) h relative to extubation. Component times had to lie within the window and span no more than 2 h. Window summaries first took the within-patient median, then medians and interquartile ranges across patients. Pre-extubation change was latest minus earliest within 72 h; slopes divided change by elapsed time in days, requiring at least two distinct measurement times. All post-extubation components preceded reintubation and death. Normalization among initially hyperosmolar patients used the latest eligible window value ≤300 mOsm/kg.

**Supplementary Results**

**Missingness and exposure timing**

The exposure and outcomes were complete by cohort definition. In MIMIC-IV, missingness was 0.7% for respiratory rate, 0.5% for oxygen saturation, 1.9% for Glasgow Coma Scale and 0.04% for creatinine; the other primary covariates were complete. In eICU-CRD, corresponding missingness was 9.9%, 1.6%, 11.9% and 0%, and all other covariates were complete (Supplementary Table S1 and Supplementary Figure S3).

The representative cOsm measurement preceded extubation by a median of 8.3 h (interquartile range, 5.3-11.2; 5th-95th percentile, 1.3-15.0) in MIMIC-IV and 8.8 h (4.9-13.1; 1.4-17.4) in eICU-CRD. In the 12-h timing sensitivity, MIMIC-IV retained 2,124 extubations with 249 events and yielded an adjusted RR of 1.49 (95% CI, 1.17-1.90). eICU-CRD retained 931 extubations with 68 events and yielded an RR of 2.06 (95% CI, 1.43-2.96). Six-hour estimates were 1.44 (95% CI, 0.97-2.13; n=790, 98 events) and 2.27 (95% CI, 1.54-3.34; n=455, 27 events) (Supplementary Tables S8-S9).

**Neighboring thresholds and cOsm categories**

For >295, >300 and >305 mOsm/kg, adjusted RRs in MIMIC-IV were 1.07 (95% CI, 0.86-1.32), 1.31 (95% CI, 1.05-1.62) and 1.21 (95% CI, 0.96-1.53). Corresponding eICU-CRD estimates were 1.39 (95% CI, 0.90-2.15), 1.88 (95% CI, 1.27-2.77) and 1.56 (95% CI, 1.01-2.40) (Supplementary Table S2 and Supplementary Figure S1).

Relative to 290-<300 mOsm/kg, MIMIC-IV RRs were 1.25 (95% CI, 0.94-1.67) for <290, 1.49 (95% CI, 1.11-2.01) for 300-<310 and 1.45 (95% CI, 1.07-1.98) for >=310 mOsm/kg. eICU-CRD estimates were 1.00 (95% CI, 0.66-1.51), 1.84 (95% CI, 1.37-2.48) and 1.91 (95% CI, 1.15-3.17), respectively (Supplementary Table S4). These categories were descriptive and were not used to select a cutoff.

**Continuous and spline analyses**

The adjusted RR per 10-mOsm/kg cOsm increase was 1.03 (95% CI, 0.96-1.11; P = 0.394) in MIMIC-IV and 1.21 (95% CI, 1.06-1.39; P = 0.006) in eICU-CRD. Per standard deviation, estimates were 1.05 (95% CI, 0.94-1.17) and 1.28 (95% CI, 1.07-1.53). At 72 h, per-10-mOsm/kg RRs were 1.03 (95% CI, 0.97-1.11) and 1.14 (95% CI, 1.02-1.28) (Supplementary Table S3).

Modified Poisson spline analyses did not support nonlinearity in MIMIC-IV (P = 0.800) or eICU-CRD (P = 0.377). Overall tests were P = 0.693 and P < 0.001, respectively (Figure 4). The MIMIC-IV continuous analyses did not provide clear evidence of a monotonic dose-response relation.

**Early death and competing-risk analyses**

Within 48 h in MIMIC-IV, the cOsm >300 group had 153 reintubations, 185 deaths without prior reintubation and 786 patients alive without reintubation. The cOsm <=300 group had 164, 182 and 1,169, respectively. At 72 h, corresponding counts were 180, 195 and 749 versus 196, 191 and 1,128. In eICU-CRD, 48-h counts were 51, 17 and 418 versus 42, 23 and 839; 72-h counts were 59, 20 and 407 versus 60, 25 and 819 (Supplementary Table S5).

At 48 h, cause-specific hazard ratios were 1.29 (95% CI, 1.02-1.63) in MIMIC-IV and 1.96 (95% CI, 1.34-2.89) in eICU-CRD. Fine-Gray subdistribution hazard ratios were 1.34 (95% CI, 1.06-1.69) and 2.03 (95% CI, 1.45-2.86). At 72 h, cause-specific estimates were 1.23 (95% CI, 0.99-1.53) and 1.62 (95% CI, 1.25-2.10), while Fine-Gray estimates were 1.29 (95% CI, 1.04-1.60) and 1.73 (95% CI, 1.37-2.17) (Supplementary Table S5 and Supplementary Figures S2 and S15).

For reintubation or death within 48 h, adjusted RRs were 1.09 (95% CI, 0.97-1.23) in MIMIC-IV and 1.42 (95% CI, 1.13-1.78) in eICU-CRD. At 72 h, estimates were 1.08 (95% CI, 0.97-1.21) and 1.28 (95% CI, 1.03-1.60). The MIMIC-IV composite was weaker than the reintubation-only association.

**Phenotype and repeated-patient sensitivity**

Excluding 40 MIMIC-IV index extubations linked to explicit unplanned-extubation labels left 2,599 extubations and 287 events; the adjusted RR was 1.30 (95% CI, 1.04-1.64). No equivalent exclusion was possible in eICU-CRD because no reliable explicit label was found. Procedure evidence was identified for 37 of 317 MIMIC-IV 48-h reintubations (11.7%) and 5 of 93 eICU-CRD reintubations (5.4%). Exclusion of admissions flagged within either 48 or 72 h left 2,593 MIMIC-IV extubations with 280 events and 1,382 eICU-CRD extubations with 88 events. Adjusted RRs were 1.32 (95% CI, 1.04-1.66) and 1.90 (95% CI, 1.25-2.89) (Supplementary Tables S6-S7).

All 2,639 MIMIC-IV extubations represented unique patients. In eICU-CRD, 1,390 extubations represented 1,334 unique patients; 41 patients contributed at least two admissions, 97 admissions came from repeated patients and 56 were beyond the first. Retaining the first eligible cOsm admission per patient left 1,334 extubations with 89 events and an adjusted RR of 1.89 (95% CI, 1.24-2.88) (Supplementary Table S8).

**Other association sensitivities**

Complete-case adjusted RRs were 1.33 (95% CI, 1.07-1.65; n=2,559, 314 events) in MIMIC-IV and 1.98 (95% CI, 1.44-2.73; n=1,114, 74 events) in eICU-CRD. Restriction to ventilation lasting at least 24 h produced estimates of 1.34 (95% CI, 1.08-1.68; n=2,542, 302 events) and 2.11 (95% CI, 1.59-2.80; n=851, 64 events) (Supplementary Table S8).

**Prognostic performance, calibration and clinical utility**

MIMIC-IV apparent AUROCs were 0.5976 for Model 0 and 0.6093 for Model 1; optimism-corrected values were 0.5785 and 0.5891. Apparent PR-AUC values were 0.1623 and 0.1654. Optimism-corrected Brier scores were 0.10521 and 0.10507.

In original fixed-coefficient eICU-CRD evaluation, Model 0 and Model 1 AUROCs were 0.5875 and 0.6074 and PR-AUC values were 0.1051 and 0.1229. Paired hospital-cluster bootstrap differences were 0.0199 (95% CI, -0.0327 to 0.0434) for AUROC, 0.0178 (95% CI, -0.0019 to 0.0342) for PR-AUC and -0.00096 (95% CI, -0.00131 to 0.00020) for Brier score (Supplementary Table S9).

Calibration intercepts were -0.91 for Model 0 and -0.89 for Model 1, calibration slopes were 0.93 and 1.14, and observed-to-expected ratios were 0.45 and 0.45. Intercept-only and intercept-plus-slope updating reduced the Model 1 Brier score to 0.06151 and 0.06145, respectively (Supplementary Figure S9). Hospital-specific performance could be estimated in only three hospitals with enough events and varied substantially (Supplementary Figure S8). Decision curves did not show a large or consistent separation between Models 0 and 1 across the prespecified threshold range (Supplementary Figure S12).

**Exploratory ACAG and component findings**

ACAG could be calculated within 24 h for 1,113 MIMIC-IV and 500 eICU-CRD extubations in the full phenotype files. The formal exploratory models were restricted to the prespecified cOsm analytical cohorts and included all 1,113 MIMIC-IV extubations and 498 eICU-CRD extubations; two eICU-CRD records with ACAG lacked calculable cOsm. Event counts were 122 and 26. In MIMIC-IV, the adjusted RR per 5-mmol/L increase was 1.01 (95% CI, 0.81-1.25; P = 0.955). In eICU-CRD, the crude RR was 0.74 (95% CI, 0.49-1.13) and the minimally adjusted Firth odds ratio was 0.69 (95% CI, 0.41-1.14). cOsm and ACAG were weakly correlated (Spearman rho = 0.145; variance inflation factor = 1.02). MIMIC-IV component RRs per standard deviation were 1.04 (95% CI, 0.94-1.15) for sodium, 0.93 (95% CI, 0.83-1.03) for glucose and 1.09 (95% CI, 0.96-1.25) for blood urea nitrogen (Supplementary Tables S10-S12 and Supplementary Figures S4-S7 and S11).

**Additional respiratory, fluid and treatment results**

Additional adjustment for P/F ratio produced little change within the corresponding measured subsets. In MIMIC-IV (1,329 extubations, 169 events), the adjusted RR was 1.47 (95% CI, 1.10-1.96) before and 1.47 (95% CI, 1.10-1.97) after P/F adjustment; corresponding eICU-CRD estimates (771 extubations, 52 events) were 1.21 (95% CI, 0.83-1.77) and 1.23 (95% CI, 0.85-1.77). FiO2/PEEP adjustment changed the same-subset RRs from 1.31 to 1.30 in MIMIC-IV and from 1.41 to 1.35 in eICU-CRD (Supplementary Tables S14-S15). Documented NIV/HFNO and the 6-h landmark analyses are reported in Supplementary Table S18.

Fluid/loop-adjusted RRs were 1.39 (95% CI, 1.10-1.75; N=2,287, 270 events) in MIMIC-IV and 3.01 (95% CI, 1.39-6.53; N=523, 34 events) in eICU-CRD. Replacing creatinine with creatinine-based AKI yielded RRs of 1.33 (95% CI, 1.05-1.67; N=2,277, 269 events) and 2.91 (95% CI, 1.43-5.92; N=521, 34 events). AKI prevalence was 735/2,629 (28.0%) and 204/1,300 (15.7%). The combined eICU-CRD respiratory/fluid model had 24 events for 14 parameters and is reported descriptively (Supplementary Tables S16-S17).

Excluding 143 MIMIC-IV admissions with identifiable hypertonic therapy retained 2,496 admissions/303 events and yielded an RR of 1.33 (95% CI, 1.07-1.66). No named hypertonic/mannitol exposure was recovered in eICU-CRD, where 24,413/114,940 medication rows lacked drug names; this did not establish absence of exposure. NIV, HFNO and either support within 6 h were documented in 83, 126 and 196 MIMIC-IV admissions and 52, 2 and 54 eICU-CRD admissions, respectively. In the no-documented-support landmark groups, RRs were 1.31 (95% CI, 0.95-1.82; N=2,069, 152 events) and 1.96 (95% CI, 1.33-2.87; N=1,295, 54 events). The supported eICU-CRD group had only six events, precluding a reliable subgroup interpretation (Supplementary Table S18).

**Exposure form and temporal findings**

The exploratory threshold was 301.964 mOsm/kg; its bootstrap median was 301.964, interquartile range 299.817-302.016 and percentile 95% range 284.151-309.063 mOsm/kg. Unchanged external application gave an RR of 2.04 (95% CI, 1.41-2.96) (Supplementary Table S19).

For effective osmolality, adjusted RRs per 10 mOsm/kg were 1.02 (95% CI, 0.93-1.11) in MIMIC-IV and 1.18 (95% CI, 0.96-1.45) in eICU-CRD (Table 3; Supplementary Table S20 and Supplementary Figure S17). The weaker effective-osmolality estimates suggest that the cOsm association was not explained by effective tonicity alone.

Pre-extubation changes overlapped between outcome groups (Supplementary Table S21). Among initially hyperosmolar patients measured within 0-24 h, normalization occurred in 126/764 (16.5%) without reintubation versus 6/87 (6.9%) with reintubation in MIMIC-IV, and 88/388 (22.7%) versus 2/20 (10.0%) in eICU-CRD. Observation stopped before reintubation or death; these comparisons are descriptive and reflect unequal measurement opportunities (Supplementary Figure S14).

**Phenotype evidence and reintubation indications**

The eICU-CRD Tier 1/2 subset comprised 835 admissions and 65 events, with an RR of 1.74 (95% CI, 1.29-2.35) (Supplementary Table S7). Explicit timed causes were available for 0/317 MIMIC-IV and 3/93 eICU-CRD reintubations; the three eICU-CRD records identified respiratory insufficiency. This coverage was insufficient for cause-specific comparisons (Supplementary Table S22).

**Supplementary Methodological References**

S1. Kidney Disease: Improving Global Outcomes Acute Kidney Injury Work Group. KDIGO Clinical Practice Guideline for Acute Kidney Injury. Kidney Int Suppl. (2012) 2:1-138.

S2. Youden WJ. Index for rating diagnostic tests. Cancer. (1950) 3:32-35. doi:10.1002/1097-0142(1950)3:1<32::AID-CNCR2820030106>3.0.CO;2-3.

S3. Fine JP, Gray RJ. A proportional hazards model for the subdistribution of a competing risk. J Am Stat Assoc. (1999) 94:496-509. doi:10.1080/01621459.1999.10474144.

**Supplementary Table Legends**

Tables S1-S22 are supplied in the accompanying workbook, with titles above each table and explanatory notes below.

**Supplementary Table S1. Covariate missingness**

RR, risk ratio; CI, confidence interval; cOsm, calculated serum osmolality. Adjusted RR models use the original eight clinical covariates and 30 core imputations, with hospital-cluster robust covariance in eICU-CRD. Additional variables are observed, not imputed.

**Supplementary Table S2. Prespecified thresholds and 72-h primary association**

RR, risk ratio; CI, confidence interval; cOsm, calculated serum osmolality. Adjusted RR models use the original eight clinical covariates and 30 core imputations, with hospital-cluster robust covariance in eICU-CRD. Additional variables are observed, not imputed.

**Supplementary Table S3. Continuous and spline analyses**

RR, risk ratio; CI, confidence interval; cOsm, calculated serum osmolality. Adjusted RR models use the original eight clinical covariates and 30 core imputations, with hospital-cluster robust covariance in eICU-CRD. Additional variables are observed, not imputed.

**Supplementary Table S4. Calculated osmolality categories**

RR, risk ratio; CI, confidence interval; cOsm, calculated serum osmolality. Adjusted RR models use the original eight clinical covariates and 30 core imputations, with hospital-cluster robust covariance in eICU-CRD. Additional variables are observed, not imputed.

**Supplementary Table S5. Early death and competing-risk analyses**

HR, hazard ratio. Cause-specific Cox uses 30 original core imputations; Fine-Gray weighted Cox uses original-core complete cases. Both use patient-cluster covariance in MIMIC-IV and hospital-cluster covariance in eICU-CRD. Binary RR, cause-specific HR and subdistribution HR are different estimands. The original eICU-CRD composite has one unavailable outcome (N=1,389), retained without imputation.

**Supplementary Table S6. Structured unplanned-extubation and procedure-related event evidence**

RR, risk ratio; CI, confidence interval; cOsm, calculated serum osmolality. Adjusted RR models use the original eight clinical covariates and 30 core imputations, with hospital-cluster robust covariance in eICU-CRD. Additional variables are observed, not imputed.

**Supplementary Table S7. Phenotype sensitivity analyses**

RR, risk ratio; CI, confidence interval; cOsm, calculated serum osmolality. Adjusted RR models use the original eight clinical covariates and 30 core imputations, with hospital-cluster robust covariance in eICU-CRD. Additional variables are observed, not imputed. The eICU-CRD strict flag identifies only one admission. Tier 1/2 restriction is a separate evidence-tier sensitivity, not manual validation. An unchanged eICU unplanned-extubation result reflects absence of reliable labels, not proven absence of unplanned events.

**Supplementary Table S8. Repeated patients and exposure timing**

RR, risk ratio; CI, confidence interval; cOsm, calculated serum osmolality. Adjusted RR models use the original eight clinical covariates and 30 core imputations, with hospital-cluster robust covariance in eICU-CRD. Additional variables are observed, not imputed. Laboratory timing summaries describe original synchronized sets; all component spreads were zero.

**Supplementary Table S9. Secondary prognostic performance and paired external differences**

Model 0 is the clinical model; Model 1 adds cOsm >300 mOsm/kg. AUROC, PR-AUC and Brier score are reported on their original scales. External intervals use 2,000 paired hospital bootstrap resamples. MIMIC-IV optimism correction applies to AUROC, Brier score and calibration, not PR-AUC or O:E; repeated PR-AUC/O:E entries in optimism-corrected rows remain apparent quantities. Component-panel confidence intervals apply only to external evaluation and are provided in S13.

**Supplementary Table S10. Exploratory ACAG analyses**

ACAG, albumin-corrected anion gap. All exposure components, including albumin, are within 24 h. Formal cOsm-cohort models use 1,113 MIMIC-IV admissions/122 events and 498 eICU-CRD admissions/26 events; 500 eICU admissions had ACAG in the broader phenotype cohort, of which two lacked cOsm. RR and OR are distinct. The eICU Firth model adjusts only for age, sex and ventilation duration. Missingness SMDs compare measured with unmeasured groups.

**Supplementary Table S11. Components and collinearity**

RR, risk ratio; CI, confidence interval; cOsm, calculated serum osmolality. Adjusted RR models use the original eight clinical covariates and 30 core imputations, with hospital-cluster robust covariance in eICU-CRD. Additional variables are observed, not imputed. Standardized components enter separate models, not a joint component model. Cross-database component results are in S20.

**Supplementary Table S12. Hospital-specific external performance**

Hospital identifiers are deidentified database codes. Hospital-specific discrimination and calibration are shown only where the original event-count rule allowed estimation; blank cells mean not estimated, not zero.

**Supplementary Table S13. Component benchmarking**

RR, risk ratio; CI, confidence interval; cOsm, calculated serum osmolality. Adjusted RR models use the original eight clinical covariates and 30 core imputations, with hospital-cluster robust covariance in eICU-CRD. Additional variables are observed, not imputed. Component units are indicated explicitly. External prediction intervals refer to fixed-coefficient evaluation, not to apparent MIMIC-IV performance.

**Supplementary Table S14. Variable definitions, sources, timing, and respiratory availability**

All respiratory measurements precede extubation and lie within 24 h. P/F pairs require arterial PaO2 and FiO2 within 1 h. FiO2 percentages are converted to fractions, not inferred from oxygen flow. Available-event counts refer to 48-h reintubation. S/F is not equivalent to P/F.

**Supplementary Table S15. Observed-subset selection and respiratory-adjusted models**

RR, risk ratio; CI, confidence interval; cOsm, calculated serum osmolality. Adjusted RR models use the original eight clinical covariates and 30 core imputations, with hospital-cluster robust covariance in eICU-CRD. Additional variables are observed, not imputed. Same-subset clinical and extended models use identical admissions. Differences from full-cohort estimates combine measurement selection with covariate adjustment.

**Supplementary Table S16. Fluid, renal, and combined model comparisons**

RR, risk ratio; CI, confidence interval; cOsm, calculated serum osmolality. Adjusted RR models use the original eight clinical covariates and 30 core imputations, with hospital-cluster robust covariance in eICU-CRD. Additional variables are observed, not imputed. C adds fluid balance and loop exposure; C2 replaces creatinine with creatinine-based AKI while retaining fluid and loop variables; D adds FiO2, PEEP, fluid and loop exposure. The eICU combined model has only 24 events for 14 parameters and is descriptive, not confirmatory. The fluid models use selected observed subsets.

**Supplementary Table S17. Creatinine-based AKI and documented drug exposures**

AKI uses in-hospital creatinine changes, without urine-output or RRT staging. eICU loop exposure denotes active orders or recorded infusions, not verified administration. No named hypertonic/mannitol exposure was recovered in eICU, but 24,413/114,940 medication rows lacked names; the unchanged exclusion estimate is not an informative negative-exposure sensitivity.

**Supplementary Table S18. Documented post-extubation support and 6-h landmark analyses**

RR, risk ratio; CI, confidence interval; cOsm, calculated serum osmolality. Adjusted RR models use the original eight clinical covariates and 30 core imputations, with hospital-cluster robust covariance in eICU-CRD. Additional variables are observed, not imputed. NIV/HFNO documentation precedes reintubation. No record is not confirmed nonuse. Landmark models include patients alive and reintubation-free at 6 h, with subsequent outcomes through 48 h. The supported eICU subgroup has 6 events for 10 parameters; its RR 16.77 (1.17-240.15) is numerically unstable and not evidence of effect modification.

**Supplementary Table S19. Exploratory derived threshold and external application**

Thresholds are mOsm/kg. Higher cOsm was fixed as the risk direction. Bootstrap resampling was at admission level. The exact derived threshold was applied unchanged externally; it is an exploratory dichotomy, not an established clinical decision threshold.

**Supplementary Table S20. Effective osmolality and standardized components**

RR, risk ratio; CI, confidence interval; cOsm, calculated serum osmolality. Adjusted RR models use the original eight clinical covariates and 30 core imputations, with hospital-cluster robust covariance in eICU-CRD. Additional variables are observed, not imputed. Effective osmolality excludes BUN. Component models are separate; estimates were not formally compared. BUN confidence limits cross 1 before rounding in eICU. Spline tests are modified-Poisson Wald tests; cOsm knots/reference are fixed at the original MIMIC-IV values.

**Supplementary Table S21. Serial cOsm, measurement coverage, and post-extubation normalization**

Serial-window summaries first take the median within each patient, then summarize across patients. Change is latest minus earliest within -72 to 0 h, requiring at least two measurements at distinct times; slope is change per elapsed 24 h. Normalization uses the latest eligible value in each post-extubation window among initially hyperosmolar patients. All components precede reintubation/death. Outcome 0 means no observed 48-h reintubation, not confirmed survival. Unequal observation time prevents causal interpretation. Semicolon triplets are median; Q1; Q3.

**Supplementary Table S22. Cross-database characteristics and explicit reintubation indications**

SMDs describe measured case-mix differences, not causal explanations for different event rates. Cause information required an explicit record within 1 h of reintubation; diagnoses, gas exchange, or subsequent treatment were not substituted. Only 0/317 MIMIC-IV and 3/93 eICU-CRD events had such records, insufficient for cause-specific analysis.

**Supplementary Figures**

**Supplementary Figure S1. Adjusted RRs for 48-h invasive reintubation at cOsm thresholds of >295, >300 and >305 mOsm/kg.**

**Supplementary Figure S2. Cumulative incidence of invasive reintubation through 72 h with death treated as a competing event.**

**Supplementary Figure S3. Covariate missingness in MIMIC-IV and eICU-CRD.**

**Supplementary Figure S4. cOsm distributions by 48-h reintubation status.**

**Supplementary Figure S5. Associations of individual cOsm components with 48-h reintubation.**

**Supplementary Figure S6. Restricted cubic spline analysis of exploratory ACAG in MIMIC-IV.**

**Supplementary Figure S7. Characteristics of patients with and without calculable ACAG.**

**Supplementary Figure S8. Hospital-specific AUROC for Model 1 in eICU-CRD hospitals with sufficient events.**

**Supplementary Figure S9. Original and recalibrated external calibration in eICU-CRD.**

**Supplementary Figure S10. Patient-level bootstrap sensitivity for the difference in external AUROC.**

**Supplementary Figure S11. Relationship between cOsm and ACAG.**

**Supplementary Figure S12. Decision-curve analysis for Models 0 and 1 using original fixed-coefficient predictions.**

**Supplementary Figure S13. Evidence-based extubation and reintubation ascertainment. Structured database-specific source rules are summarized; these are not manual adjudication. See Supplementary Methods, Event definitions and source implementation.**

**Supplementary Figure S14. Serial cOsm by 48-h reintubation status. Points are medians of patient-specific window medians; bars are interquartile ranges. Post-extubation observations stop before reintubation or death. No observed reintubation does not imply survival. Window-specific denominators and normalization are in Supplementary Table S21.**

**Supplementary Figure S15. Cumulative incidence of reintubation with death as a competing event. Exposure groups are cOsm >300 and ≤300 mOsm/kg. Adjusted competing-risk estimates are reported separately in Supplementary Table S5.**

**Supplementary Figure S16. Distribution of the exploratory MIMIC-IV threshold across 1,000 admission-level bootstrap resamples. The derived threshold was applied unchanged to eICU-CRD (Supplementary Table S19).**

**Supplementary Figure S17. Restricted cubic spline associations of pre-extubation effective osmolality with 48-h invasive reintubation. Effective osmolality was calculated as 2 × sodium + glucose/18, excluding BUN. Curves show adjusted relative risks with 95% confidence intervals. The same spline specification and reference value were applied across cohorts. The common reference was 287.444 mOsm/kg, the MIMIC-IV median, with knots at 275.278, 287.444 and 302.178 mOsm/kg. Panel-specific overall and nonlinear P values are shown. Both panels use identical axis scales; the horizontal line denotes RR = 1. No effective-osmolality threshold is imposed (Supplementary Table S20).**
